# A randomized crossover study of cardiologist reporting of severe aortic stenosis with/without assistance from artificial intelligence (AI)

**DOI:** 10.1016/j.isci.2026.115562

**Published:** 2026-04-02

**Authors:** David Playford, Dane Brescacin, Chris Frampton, Naveed Younis, Geoff Strange

**Affiliations:** 1 The University of Notre Dame, Fremantle, WA, Australia; 2 Echo IQ, Sydney, NSW, Australia; 3 University of Otago, Christchurch, New Zealand; 4 St Bernard’s Hospital, Jonesboro, AR, USA; 5 University of Sydney, Sydney, NSW, Australia

**Keywords:** Health sciences, Medicine, Medical specialty, Cardiovascular medicine, Health technology

## Abstract

Inaccurate echocardiographic diagnosis of severe aortic stenosis (AS) may be improved by AI. A randomised case-control fully-crossed, paired-reader, paired-case (MRMC) study evaluated the performance of AI in assisting cardiologist reporting of severe AS. 200 echos were independently adjudicated by expert cardiologists, 100 with severe AS (cases) and 100 without severe AS (controls). The AI was then applied and correctly classified all (100%) cases of severe AS, and 18 (36%) of moderate AS with signs of cardiac damage. Five independent cardiologists reported all echos according to the MRMC protocol. Without AI, cardiologists frequently misdiagnosed low-gradient severe AS. With AI, AS diagnosis rates were not improved, but AI improved reader concordance, referral for further investigation and reporting time. Cardiologists frequently misdiagnose low gradient severe AS, whereas measurement AI showed excellent accuracy. Lack of improvement in cardiologist accuracy with AI assistance highlights opportunities to improve trust in AI.

## Highlights

• We performed an RCT to test whether cardiologists report severe AS better with AI assistance
• 200 echos (from no AS to severe AS) were diagnosed by an expert panel, then AI was applied
• Echos were randomized, and the AS severity was reported by five independent cardiologists
• The AI outperformed cardiologists, but cardiologists were reluctant to take the AI’s advice

## Graphical abstract

**Figure:**
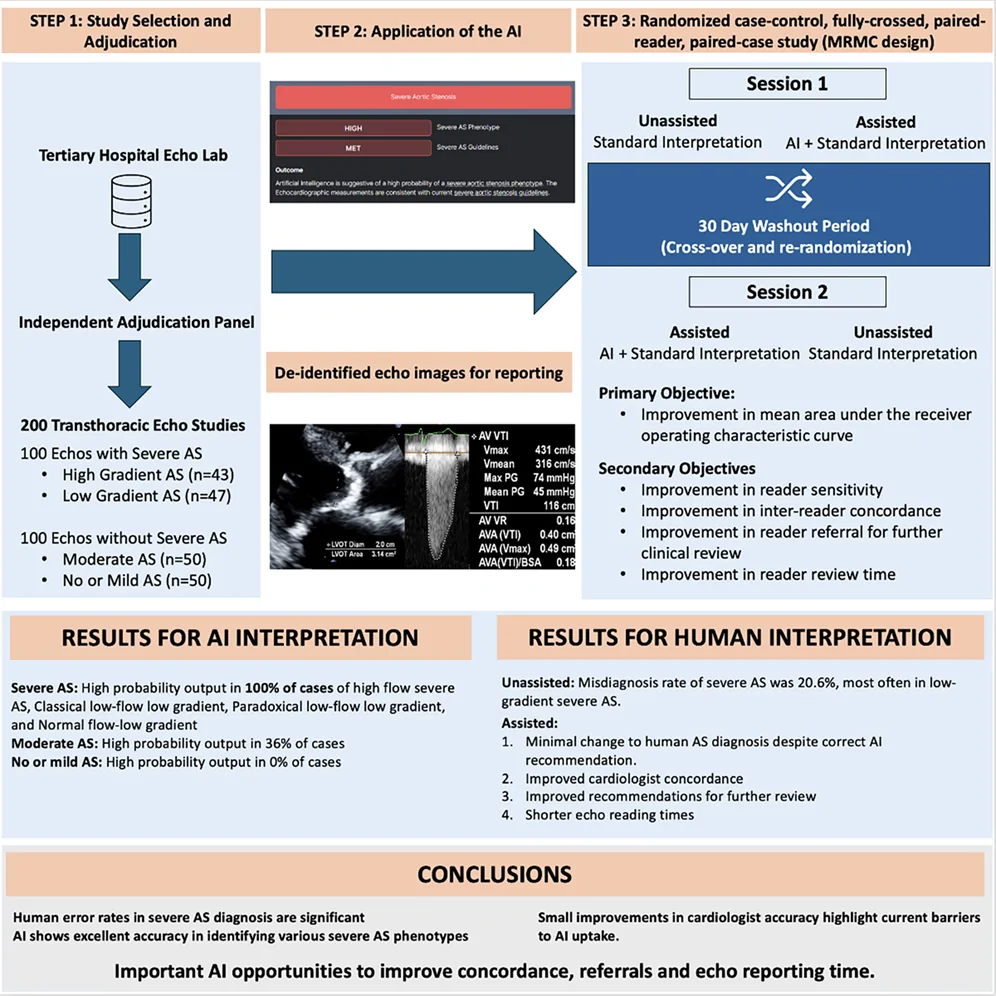

## Introduction

Severe aortic stenosis (AS) is the commonest valve lesion requiring intervention, particularly in older individuals.^1^ Due to substantial societal cost and high mortality associated with untreated severe AS,^2^^,^^3^ particularly in the presence symptoms,^4^ early and accurate diagnosis and prompt referral for the consideration of valve therapy^5^^,^^6^ is essential. Echocardiography remains pivotal to AS diagnosis (based predominantly on transvalvular gradients and calculated aortic valve area), although AS subtypes such as low-gradient AS (including low-flow low-gradient and paradoxical low-gradient) pose unique diagnostic challenges,^7^^,^^8^ leading to frequent under-diagnosis and under-treatment.^9^^,^^10^^,^^11^

Since the impetus for clinical review, further investigation, and consideration of valve therapy is driven by the conclusions on the transthoracic echocardiogram report,^12^ diagnostic variability (predominantly under-diagnosis) may adversely impact valve team referrals, intervention rates, and mortality risk. These observations have prompted calls for clinical decision support systems in echocardiography.^13^^,^^14^

To assist cardiologists in AS diagnosis, we developed an automated AI system which uses previously obtained echocardiographic measurement data (without the need for image recognition) to identify^15^ severe AS phenotypes, along with severe AS guideline recommendations.^1^^,^^16^^,^^17^^,^^18^ This system automatically identifies the complex echocardiographic patterns known to be associated with progressive AS, including pathophysiologic changes in cardiac structure/function that may be associated with different patterns such as low-gradient AS.^19^^,^^20^^,^^21^ The use of AI in echocardiography has been the focus of considerable recent research and development,^22^ including new methods of diagnosis of valvular heart disease using image recognition,^15^^,^^23^^,^^24^^,^^25^^,^^26^^,^^27^ although the approach we have taken to detect disease phenotypes using only echocardiographic measurements remains unique.^12^^,^^16^^,^^18^^,^^28^^,^^29^

**Figure 1:**
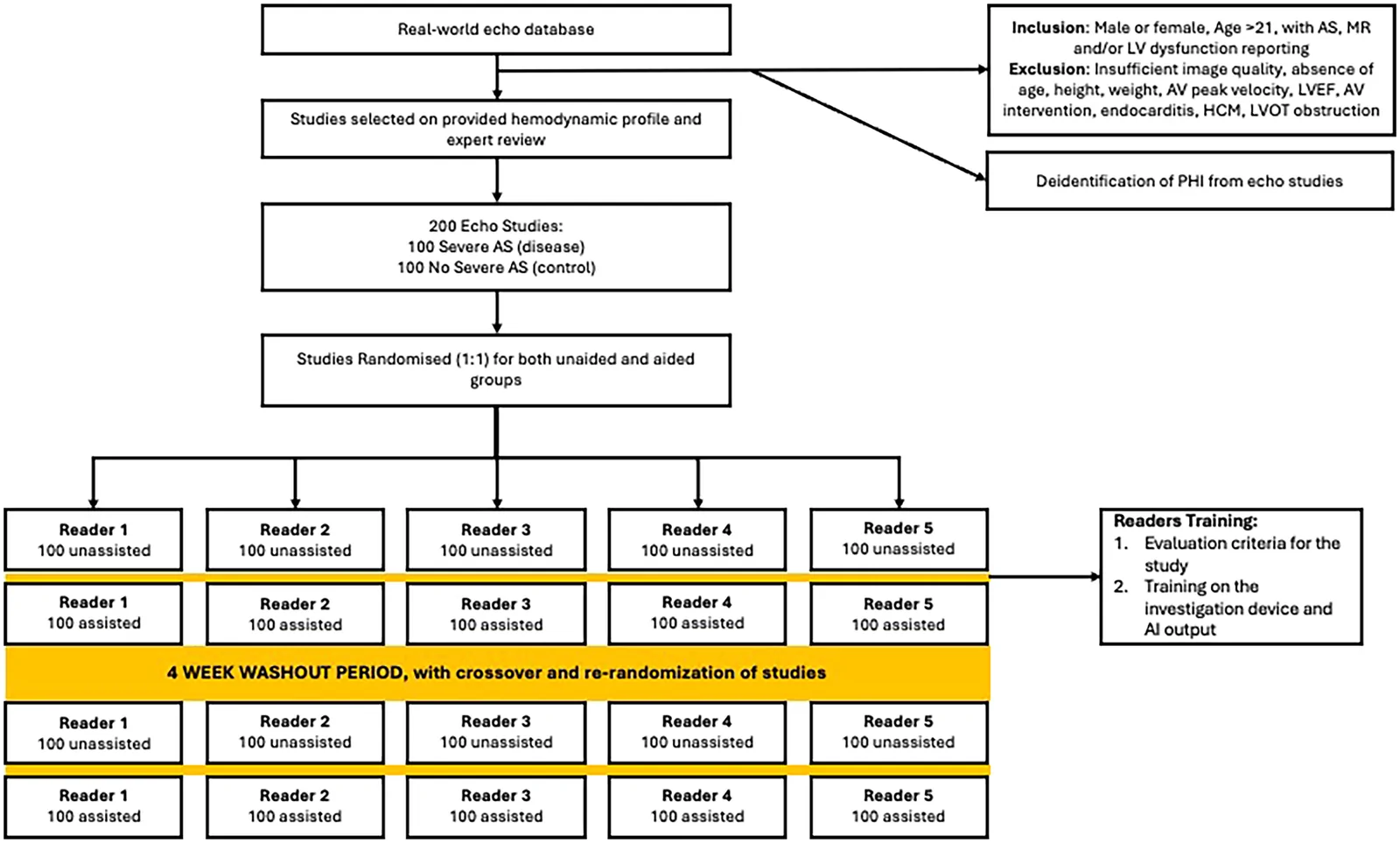
Study flow chart The echocardiography laboratory at the study site was interrogated, and 200 individual patients meeting inclusion criteria (100 cases with severe AS, and 100 controls without severe AS but having an additional diagnosis of either ≥ moderate MR and/or ≥ moderate LV dysfunction) were selected by an adjudication panel. The AI was then applied after the final TTE interpretation. Randomization was undertaken, and five US board-certified cardiologists from the same tertiary hospital cardiology department, with no prior knowledge or exposure to the AI (to avoid any initial reporting bias), participated in the study. A sixth reader was initially involved in the study but was subsequently withdrawn by the site due to a major protocol deviation. Initial training intentionally did not include information or training on the AI, which was provided after the first 100 reads. For the remaining 100 reads, the results of the AI were revealed along with the images. After a four-week washout period and re-randomization, the 200 studies were read again independently by each cardiologist, half with the assistance of the AI.

No prior randomized controlled clinical trials have been performed to examine the impact of the application of AI to assist the human diagnosis of various patterns of AS. We therefore designed a multi-reader multi-case randomized controlled study (see Figure 1) to test whether AI could improve clinical echocardiographic reporting of AS. We hypothesized that AI assistance provided at the time of echocardiographic reporting could improve diagnostic accuracy.

## Results

### Study population

A total of 200 echos were included in the study, shown in Table 1, with an overall mean age (± standard deviation, SD) of 73.55 ± 12.50 years and 50.5% (*n* = 101) females. 86.5% (*n* = 173) of the study population were of white ethnicity, with the remainder being African American (10.5%, *n* = 21), Hispanic or Latino (2.5%, *n* = 5), and a single patient with undisclosed ethnicity.

**Table 1:** Characteristics of the study population (*n* = 200)

	Age, years	Female sex	LVEF (%)	Mean Aortic Gradient (mmHg)	Peak Aortic Velocity (m/s)	Aortic Valve Area (cm^2^)	AI high probability output (%)
**High Gradient Severe AS (*n* = 47)**	74.53 (9.54)	53.2%	59.82 (13.00)	47.31 (13.47)	434.16 (28.16)	0.80 (0.26)	47 (100%)
**Low Gradient Severe AS (*n* = 53)**	76.94 (9.34)	52.8%	50.39 (17.53)	22.08 (8.02)	305.77 (59.83)	0.78 (0.14)	53 (100%)
Classical Low Flow Low Gradient (*n* = 19)	78.58 (9.22)	52.6%	34.97 (14.05)	19.58 (9.65)	282.84 (73.96)	0.74 (0.14)	19 (100%)
Paradoxical Low Flow Low Gradient (*n* = 20)	77.30 (7.73)	40.0%	59.38 (12.00)	24.05 (7.25)	318.05 (51.27)	0.78 (0.14)	20 (100%)
Normal Low Flow Low Gradient (*n* = 14)	74.21 (11.49)	71.4%	59.13 (12.62)	22.64 (5.99)	319.36 (41.28)	0.85 (0.13)	14 (100%)
**Moderate AS** (*n* = 50)	76.32 (12.39)	50.0%	58.84 (12.32)	16.43 (7.16)	269.31 (57.26)	1.15 (0.16)	18 (36%)
**No or Mild AS** (*n* = 50)	66.26 (15.08)	46.0%	34.72 (12.84)	5.21 (2.44)	156.50 (112.87)	1.39 (0.62)	0 (0%)

Data presented as mean (standard deviation, SD) except for sex, where percent female is displayed. High-gradient severe AS was defined as a peak aortic velocity between 4.0 and 4.5 m/s and/or a mean transaortic gradient of between 40 and 45 mmHg. Low-gradient severe AS was defined as an AVA ≤1.0 cm^2^ and both a peak aortic velocity between <4.0 m/s and a mean transaortic gradient of between <40 mmHg. Three subtypes of low-gradient severe AS were included: Classical low flow low gradient severe AS had the additional characteristic of LVEF <50% and stroke volume index [SVi] < 35 mL/m^2^, whereas paradoxical low flow low gradient severe AS had LVEF ≥50% and stroke volume index [SVi] < 35 mL/m^2^, and normal flow low gradient severe AS had stroke volume index [SVi] < 35 mL/m^2^. AI high probability output refers to the EchoSolv AS output indicating a high probability of the severe AS phenotype. LVEF = left ventricular ejection fraction. AI = artificial intelligence.

Of the 100 echos with severe AS (cases), 47 had high-gradient severe AS (mean AVA 0.80 ± 0.26 cm^2^, peak velocity 4.34 ± 0.28 m/s, mean gradient 47.31 ± 31 mmHg), and 53 had low gradient severe AS (mean AVA 0.78 ± 0.14 cm^2^, peak velocity 3.05 ± 0.59 m/s, mean gradient 22.08 ± 8.02 mmHg). The low-gradient severe AS groups were further sub-classified into classical low-flow low-gradient severe AS (*n* = 19, mean AVA 0.74 ± 0.14 cm^2^, peak velocity 282.84 ± 73.96 m/s, and mean gradient 19.58 ± 9.65 mmHg), paradoxical low-flow low-gradient severe AS (*n* = 20, mean AVA 0.78 ± 0.14 cm^2^, peak velocity 318.05 ± 51.27 m/s, and mean gradient 24.05 ± 7.25 mmHg), and normal-flow low-gradient severe AS (*n* = 20, mean AVA 0.85 ± 0.13 cm^2^, peak velocity 319.36 ± 41.28 m/s, and mean gradient 22.64 ± 5.99 mmHg). The 100 echos without severe AS (controls) included 50 with moderate AS (most also having evidence of cardiac damage) and 50 with either mild or no AS, and the presence of left ventricular (LV) systolic dysfunction (LVEF <40%) and/or progressive or severe mitral regurgitation.

The AI correctly identified severe AS in all (*n* = 100) cases, including the low-gradient AS cases (see Table 1). 36% of the moderate AS cases were also identified as high probability by the AI, where evidence of cardiac damage was also present. Zero cases of either no or mild AS were identified as high probability by the AI. When initially blinded to both the purpose of the study and the AI (*n* = 100 cases), cardiologists misdiagnosed severe AS between 6 and 54% of the time, showing an overall error rate of 20.6%. Cardiologist errors were less common in high-gradient severe AS (sensitivity 94.0%, 95% CI 90.2–96.7%, AUROC 0.94, 0.92–0.96) than with low-gradient AS (sensitivity 66.4%, 95%, 95% CI 60.4%–72.1%, AUROC 0.80, 0.76–0.84).

### AI guidance during echocardiogram reading

When AI guidance was provided to the cardiologist at the time of echocardiogram reading, no cardiologist consistently accepted the recommendation of the AI. In general, improvement in cardiologists' accuracy paralleled the acceptance of AI guidance. Two cardiologists showed a statistically significant improvement in their accuracy, with no significant improvement in the remaining three readers. The improvement in sensitivity was less obvious in high-gradient AS which was already high at baseline (assisted sensitivity 97.4%, 95% CI 94.5–99.1%, AUROC 0.95, 0.93–0.97), than the improvement in sensitivity observed low-gradient AS (assisted sensitivity 71.7%, 95% CI 65.9–77.0%, AUROC 0.82, 0.79–0.86), although neither improvement was statistically significant (most likely due to the small number of cases in each group).

**Figure 2:**
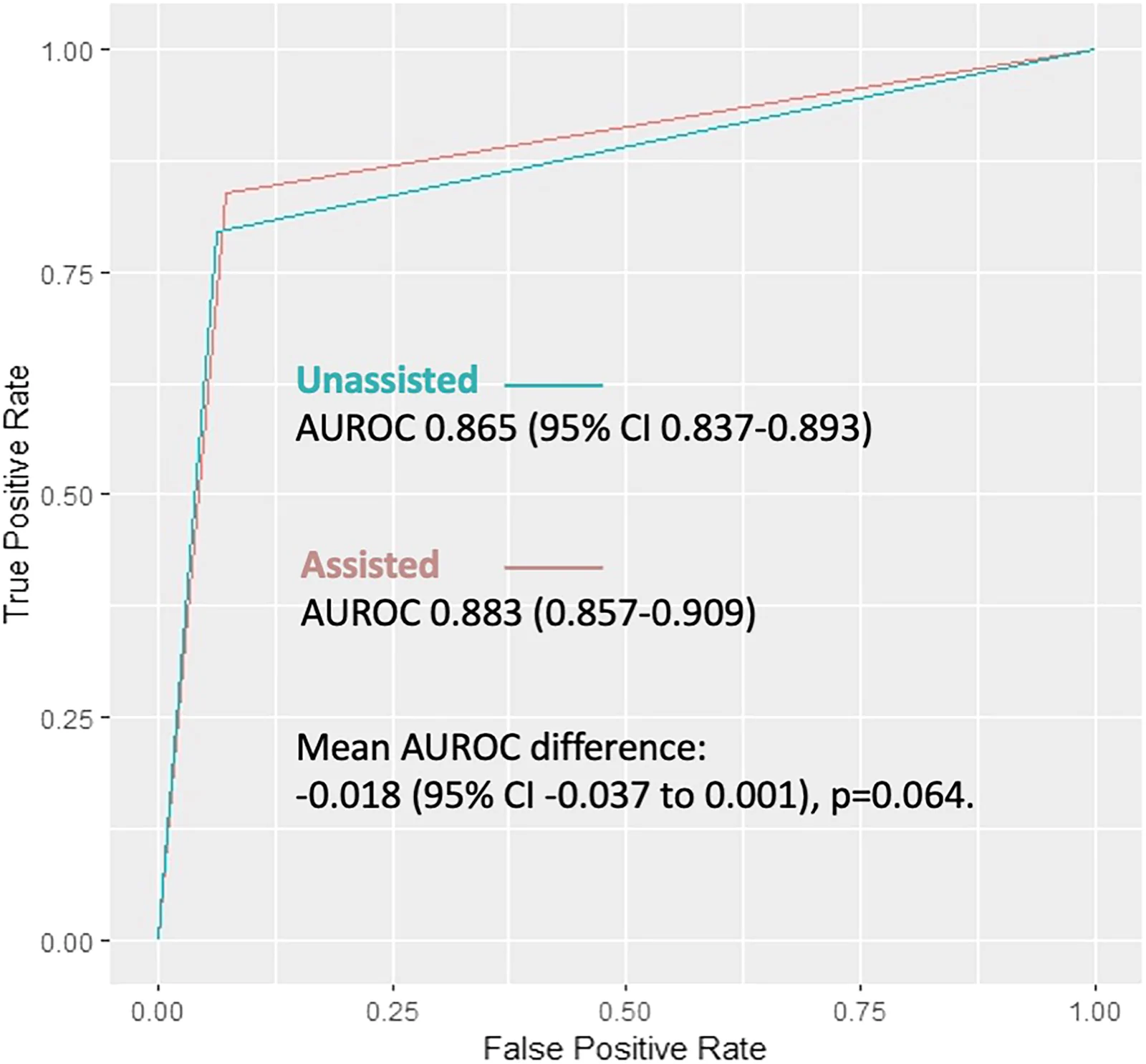
Receiver operating characteristic (ROC) curves demonstrate the accuracy of severe AS diagnosis for both unassisted and AI-assisted echocardiographic review Pooled (5 readers) ROC curves for AI-assisted vs. unassisted accuracy in diagnosing severe AS. The AI-assisted reads are shown in red, and the unassisted reads are in blue.

The primary outcome of the study (pooled analysis of the change in mean AUROC assisted vs. unassisted diagnosis of severe AS) did not meet statistical significance, shown in Table 2 and Figure 2, with a mean AUROC difference of 0.018 (95% CI −0.001 to 0.037), *p* = 0.064. The AUROC for unassisted diagnosis of severe AS was 0.865 (95% CI 0.837–0.893), compared with the mean AUROC for assisted diagnosis of severe AS of 0.883 (0.857–0.909).

**Table 2:** The primary and secondary study endpoints with and without AI assistance

Study Endpoints		AI assistance	Pooled mean (95% CI)	Pooled Difference (95% CI)	*p*-value
Primary endpoint (AUROC)		Unassisted	0.865 (0.837–0.8293)	0.018 (−0.001–0.037)	*p* = 0.064
		Assisted	0.883 (0.857–0.909)		
Secondary endpoints	Sensitivity (%)	Unassisted	79.4% (75.9–82.9%)	4.4% (−0.4–9.2%)	*p* = 0.073
		Assisted	83.8% (80.6–87.0%)		
	Inter-reader concordance (Cohen’s kappa)	Unassisted	0.641 (0.597–0.685)	0.027	
		Assisted	0.667 (0.623–0.711)		
	Referral rates for further investigation (AUROC)	Unassisted	0.787 (0.758–0.816)	0.036 (0.007–0.065)	*p* = 0.016
		Assisted	0.823 (0.797–0.849)		
	Reading times (minutes)	Unassisted	5.970 (5.769–6.178)	1.440 (24.0% decrease)	*p* < 0.001
		Assisted	4.530 (4.377–4.688)		

The primary endpoint is the pooled reader trapezoidal area under the receiver operating characteristic (AUROC) curve, with 95% confidence intervals (CI), followed by the pairwise difference in AUROC (with 95% CI), and associated *p*-value. For secondary endpoints, the sensitivity is provided as a percentage (with 95% CI), inter-reader concordance as the kappa score, referral for further investigation as AUROC (95% CI), and reading times as the number of minutes per study (95% CI).

Secondary outcomes (Table 2) included reader sensitivity in the diagnosis of severe AS, which non-significantly improved with AI assistance from 79.4% (75.9–82.9%) to 83.8% (80.6–87.0%), a mean improvement of 4.4% (−0.4 to 9.2%, *p* = 0.073). There was a 21.4% decrease in incorrect severe AS diagnosis rates for AI-assisted vs. unassisted reads. Each failure to improve the accuracy of reads was accompanied by a lack of concordance (agreement) with the AI guidance. Similarly, reader concordance did not significantly improve with AI assistance, either in high-gradient (mean kappa improvement kappa = 0.031) or low-gradient (mean kappa improvement kappa = 0.012). When each cardiologist was asked whether, in their opinion, the patient with severe AS should be referred for further evaluation (including provocative testing such as dobutamine or exercise stress echocardiography, clinical cardiology review and/or heart team evaluation if appropriate), there was improved uniformity of referral patterns with AI assistance (AUROC 0.787, 95% CI 0.758–0.816 without assistance, and 0.823, 0.797–0.849, respectively, with AI assistance), corresponding to an overall improvement of 0.036, z-interval 2.406, *p* = 0.016. There was a substantial decrease in echocardiogram review times with the assistance of the AI, with all cardiologists showing an improvement ranging from 11.8 to 32.0% and an overall review time improvement of 24.1% (*p* < 0.001). This shortened review time was similar for both groups: high-gradient (absolute difference 1.47 min, percent reduction 24.5%, *p* < 0.001), and low-gradient (absolute difference 1.51 min, percent reduction 24.8%, *p* < 0.001).

### Reporting habits and AI resistance

To establish whether long-standing echocardiogram reporting habits may have impacted the choice whether or not to follow the AI recommendation, we inspected performance metrics of each reading cardiologist. One cardiologist with >5 years since board certification had low unassisted AUROC and sensitivity, which remained unchanged after AI assistance, suggesting resistance to change in echocardiogram reporting practice. Similarly, the choice of referral for further investigation did not alter throughout the study for this cardiologist. For the remaining reading cardiologists, a post-hoc exploratory analysis was performed (Table 3), demonstrating a significant improvement in the AUROC from 0.866 (95% CI 0.838 to 0.894) without assistance to 0.894 (0.868 to 0.920) with assistance, *p* = 0.005. This was accompanied by a 5.5% (95% CI 0.0–11.0%, *p* = 0.05) improvement in severe AS diagnosis sensitivity, higher referral rates (*p* = 0.002), faster reading times (*p* < 0.001), and improved inter-reader concordance (*p* = 0.009).

**Table 3:** Post-hoc analysis of four readers, excluding the single reader whose accuracy worsened with AI assistance (see text for full explanation)

Study Endpoints		AI assistance	Pooled mean (95% CI)	Pooled Difference (95% CI)	*p*-value
Primary endpoint (AUROC)		Unassisted	0.866 (0.838–0.894)	0.028 (0.008–0.047)	*p* = 0.005
		Assisted	0.894 (0.868–0.920)		
Secondary endpoints	Sensitivity (%)	Unassisted	77.8% (73.7–81.8%)	5.5% (0.0–11.0%)	*p* = 0.05
		Assisted	83.3% (79.6–86.9%)		
	Inter-reader concordance (Cohen’s kappa)	Unassisted	0.630 (0.573–0.687)	0.067	–
		Assisted	0.697 (0.641–0.754)		
	Referral rates for further investigation (AUROC)	Unassisted	0.769 (0.739–0.799)	0.051 (0.019–0.083)	*p* = 0.02
		Assisted	0.820 (0.793–0.847)		
	Reading times (minutes)	Unassisted	5.850 (5.618–6.093)	1.575 (26.9% decrease)	*p* < 0.001
		Assisted	4.275 (4.105–6.093)		

The primary and secondary study endpoints with and without AI assistance. The primary endpoint is the pooled reader trapezoidal area under the receiver operating characteristic (AUROC) curve, with 95% confidence intervals (CI), followed by the pairwise difference in AUROC (with 95% CI), and associated *p*-value. For secondary endpoints, the sensitivity is provided as a percentage (with 95% CI), inter-reader concordance as the Cohen’s kappa score, referral for further investigation as the AUROC (95% CI), and reading times as the number of minutes per study (95% CI).

### High-gradient and low-gradient AS and reader accuracy

A further secondary analysis was performed to identify whether high- or low-gradient AS was associated with greater reader inaccuracy. For high-gradient AS, all cardiologists showed high diagnostic accuracy in their unassisted reads, ranging from 0.898 to 0.985, which did not improve significantly with the addition of AI guidance. The review time became significantly shorter (mean 6.0 min unassisted to 4.5 minutes assisted, a 24.5% improvement, *p* < 0.001) with AI guidance in high-gradient AS, but other performance metrics remained similar with and without AI assistance. In contrast, low-gradient AS was more variable, with lower baseline accuracy (AUROC 0.800, 95% CI 0.762 to 0.839) than for high-gradient AS. Four cardiologists improved their AUROC with AI assistance in the low-gradient AS group, and one cardiologist (also referred to above) showed a decrease in accuracy from unassisted to assisted interpretation of low-gradient AS. All cardiologists improved their reading time with AI assistance, from 6.103 (95% CI 5.859 to 6.357) to 4.588 (4.404 to 4.779) minutes, *p* < 0.001.

## Discussion

To our knowledge, this is the first randomized controlled study in AS examining the effect of AI assistance on human behavior during routine echocardiographic reporting. 200 echocardiograms, half of which had severe AS (including high- and low-gradient cases), were first reported by a panel of experts and then analyzed by the AI system. The AI correctly allocated a high probability classification to each case of severe AS, as well as highlighting some cases of moderate AS (with signs of cardiac damage), whereas no patients with ≤mild AS were highlighted by the AI. Five board-certified cardiologists, initially blinded to the purpose of the study and to the AI, read each echocardiogram (images and measurements) as part of standard clinical workflow, with severe AS misdiagnosis rates of 20.6%. After unblinding and with the assistance of the AI, the primary outcome (change in mean AUROC assisted vs. unassisted diagnosis of severe AS) did not significantly improve, whereas the consistency, echocardiogram reading times, and referral patterns for further evaluation of severe AS all improved significantly, although misdiagnosis rates improved only slightly. Most physician errors were in low-gradient severe AS, and these errors persisted despite prompting from the AI as to the correct diagnosis.

Humans may hesitate to incorporate an AI diagnosis in echocardiography studies due to a lack of trust in the AI’s decision-making process, fearing that it may overlook subtle abnormalities or misinterpret complex patterns that rely on context and experience. However, the AI was intentionally developed to automatically identify the relationships between echocardiographic measurements to predict the phenotype associated with severe AS.^16^ More recently, we refined the algorithm by applying newer machine learning techniques and clinical practice guideline criteria,^1^^,^^17^^,^^18^ and showed that the complex echocardiographic patterns known to be associated with progressive AS, and identified by an experienced cardiologist, are also identified by the AI system. Such pathophysiologic changes include AS-specific abnormalities such as aortic valve calcification, restricted cusp motion, and elevated transvalvular gradients, and other non-valvular changes typically found in severe AS, such as LV hypertrophy, diastolic dysfunction, increased left atrial pressure and volume, atrial fibrillation, and pulmonary hypertension.^19^^,^^20^^,^^21^ We identified higher human error rates in low-gradient than in high-gradient AS that could be corrected by the AI, a finding consistent with previous studies^12^ and the frequent accompaniment of low-gradient AS with comorbidities such as hypertension, diabetes, chronic kidney disease and coronary artery disease. Identification of these cardiac damage^30^^,^^31^ changes may improve timely identification of individuals in need of AV replacement, with the goal of improving clinical outcomes and minimizing mortality risk.^32^

Recent data have challenged the human-only approach to echocardiographic AS diagnosis. Poor patient outcomes are not limited only to severe symptomatic AS,^33^^,^^34^ and symptoms are often associated with cardiac damage and comorbidities rather than aortic valve obstruction.^35^ In addition, as highlighted by Li and others, current traditional approaches result in under-reporting and undertreatment of severe AS, despite these patients meeting current guideline recommendations for the consideration of valve intervention.^9^^,^^10^^,^^11^ Humans are more likely to correctly diagnose high-gradient than low-gradient AS, and possibly males rather than females, due to the smaller aortic valve area at a similar transvalvular gradient.^12^ These limitations, as well as advances in transcatheter aortic valve intervention (TAVI),^36^ including new indications in both symptomatic and asymptomatic severe AS,^37^ have led to calls for improvements in physician diagnosis of AS through the implementation of AI into echocardiogram reporting.^13^^,^^14^ Finally, the AI identification that some patients with moderate AS had a high probability of the severe AS phenotype is consistent with previous studies, where specific risk phenotypes are associated with a higher risk of death and may benefit from timely clinical review.^4^^,^^38^^,^^39^^,^^40^^,^^41^^,^^42^^,^^43^

Although not statistically significant, we found that less experienced cardiologists appeared to be more amenable to the assistance of the AI compared with their more experienced colleagues. We hypothesize that well-entrenched reporting habits are slow to change with AI assistance, and a dynamic approach to education and familiarization to enhance cardiologist engagement may be needed prior to clinical deployment (including clinical outcome data, model explainability, reliability, and the potential for bias).

In summary, in this randomized, blinded crossover study that examined the effect of AI assistance on human behavior during echocardiographic reporting of AS, we demonstrated excellent accuracy of the AI system to identify severe AS (using measurement data alone, no image analysis), correctly identifying all independently adjudicated cases. Prior to AI exposure, in this selected cohort, the human-alone misdiagnosis of severe AS was 20.6%, predominantly in reporting of low-gradient AS. After AI-assisted clinical decision support was provided, human reporting patterns remained similar, particularly in the most experienced cardiologists, suggesting well-established reporting patterns that were not influenced by the assistance of the AI. However, reader concordance, agreement on referral for review/testing, and reporting times all favored AI-assisted echocardiogram reporting. This study highlights the importance of AI to improve physician accuracy, efficiency, and consistency in echocardiography reporting, and the need for a dynamic approach to ensure cardiologist engagement prior to clinical deployment.

### Limitations of the study

There are several limitations of this study. First, we did not prospectively enroll patients based on their AI-assisted echocardiographic report, and thus, patient management was not altered. New prospective studies are being designed to examine the effect of AI-enhanced echocardiogram reporting on patient outcomes. Second, we selected patients meeting specific inclusion and exclusion criteria, which may not reflect the use of AI in standard clinical practice in a real-world environment. Third, we relied on the review of independent expert cardiologists to assign each severe AS diagnosis. Although a misdiagnosis of severe AS at this stage is possible, the study design required concordance of at least two cardiologists in the diagnosis, with an adjudication meeting held with a third cardiologist if any disagreement was found. Despite this rigorous approach, we acknowledge the difficulty in assigning a definitive severity classification from a transthoracic echocardiogram alone in some patients. Fourth, it can be clinically challenging to separate true severe AS from pseudo-severe AS due to non-severe AS in the setting of a low cardiac output. We did not provide additional studies such as dobutamine stress echocardiography, cardiac MRI, transesophageal echocardiography, or cardiac catheterization, nor did we provide additional clinical information. Acknowledging that these data may be informative in a clinical setting and assist in decision-making, we requested the reporting cardiologists to highlight patients needing further investigation, clinical review, and/or referral to the heart valve team. Finally, the five US board-certified cardiologists interpreting the study were from the same US tertiary hospital cardiology department, which could potentially limit the generalizability of these findings to other regions and centers where AI receptivity may be higher.

## Resource availability

### Lead contact

Professor David Playford, david.playford@echoiq.ai.

### Materials availability

Study protocol and statistical analysis plan will be available upon request. This study did not generate new unique reagents.

### Data and code availability

• Data: Individual participant data that underlie the results reported in this article will be available (including text, tables, figures, and appendices) following a data access request to the lead contact, and a data access agreement signed by both parties. The data will be available from 3 months to 36 months post article publication.
• Code: This paper does not report original code.
• Other items: Any additional information required to reanalyze the data reported in this paper are available from the lead contact upon request. The AI product (EchoSolv AS, Echo IQ, ASX:EIQ) is regulatory cleared in the USA and is intended for patients who 18 years or older who have an echocardiogram performed as part of routine clinical care. Outside the USA, the product may not be regulatory cleared for clinical use.

## Acknowledgments

This study was funded by a Heart X technology accelerator grant from the Saint Bernard Medical Centre (Jonesboro, Arkansas, USA), and sponsored by Echo IQ (ASX: EIQ).

## Author contributions

D.P. – study design and oversight, results interpretation, and primary responsibility for manuscript preparation. D.B. – study design and oversight, Liaison with Clinical Trials Organization, results interpretation, and manuscript preparation. C.F. – independent statistical analysis, interpretation of results, and manuscript preparation. N.Y. – site principal investigator, study oversight, interpretation of results, and manuscript preparation. G.S. – study concept, overall study design and oversight, coordination of the study team, results interpretation, and manuscript preparation.

## Declaration of interests

D.P. – Consulting fees and travel assistance from Echo IQ. No other conflicts. D.B. – Employee of Echo IQ. C.F. – No conflicts declared. N.Y. – No conflicts declared. G.S. – Consulting fees and travel assistance from Echo IQ. No other conflicts.

## STAR★Methods

### Key resources table

**Table:** 

REAGENT or RESOURCE	SOURCE	IDENTIFIER
**Software and algorithms**		

EchoSolv AS software	Echo IQ Ltd (ASX:EIQ)	Not Applicable

### Experimental model and study participant details

#### The National Echo Database of Australia (NEDA)

NEDA contains the echocardiographic measurement and report data from adult (age ≥18 years) men and women undergoing echocardiography for clinical indications^44^ to investigate or follow-up cardiac disease. It conforms to the ethical standards of the Declaration of Helsinki^45^ and the reporting of routinely collected observational and outcome data as outlined by the RECORD Statement.^46^ NEDA is registered with the Australian New Zealand Clinical Trials Registry (*ACTRN12617001387314*). Ethical approvals have been obtained from all relevant Human Research Ethics Committees (principal ethics application *HREC/15/RPAH/530*). Australia is highly a multicultural nation, and the second iteration of the NEDA captured data from 23 centers Australia-wide collected from January 1, 2000 to May 21, 2019. As described in greater detail previously^44^^,^^47^^,^^48^ and without any form of reporting bias, NEDA collects and synthesises routine echocardiogram reporting data using a standardised protocol to ensure that all data from participating sites were cleaned and transformed into standard NEDA format to generate uniform echocardiogram profiling data and to remove duplicate and/or impossible measurements or investigation. A de-identified version of NEDA was then used to create the AI models.

#### Development of the AI model

We have previously reported the capacity for AI (EchoSolv AS, Echo IQ Ltd, Sydney, Australia ASX:EIQ) to enhance the detection of severe AS, initially with the development of the system using data from 442,824 individuals from NEDA.^16^^,^^18^ Briefly, the first development step was the creation of an imputation model, which created a complete set of echocardiographic data that was then used for further model development. The accuracy of this imputation model has been previously published.^18^ The second step was to then use all available echocardiographic measurement data (up to 280 possible individual echocardiographic variables, representing base measurements and calculations as part of a standard echocardiography examination), to train a mixture density neural network to predict an aortic valve area (AVA) ≤1.0 cm^2^. The unique method of training specifically *excluded* all left ventricular outflow tract velocity/gradient or dimension/area measurements, allowing the AI to only see the aortic velocity measurements and the remainder of echocardiographic measurement data, thereby forcing the AI to predict the probability of an AVA ≤1.0 cm^2^ by identifying the measurements that typically accompany severe AS (indicating “cardiac damage” such as left ventricular hypertrophy, impaired relaxation, signs of elevated filling pressure, LV systolic dysfunction, left atrial enlargement and pulmonary hypertension).^30^ This strategy offered the major advantage of correctly identifying the AS risk phenotype, including AS subtypes typically challenging for cardiologists (such as low-gradient severe AS). The optimal probability threshold for severe AS detection was identified at the F1 score probability of 0.235, above which is designated “high probability.” An additional probability threshold was identified between 0.0625 and 0.235, indicating a “medium probability,” with low probability designation for probability outputs below 0.0625. A further feature included the automatic application of clinical practice guidelines (mean aortic gradient ≥40 mmHg, peak aortic velocity ≥4.0 m/s, and/or aortic valve area ≤1.0 cm^2^).^1^^,^^17^ When independently applied to 184 301 individuals, the area under receiver operating characteristic curve for the AI-DSA to detect severe AS was 0.986 (95% CI 0.985 to 0.987). Subsequently, we externally validated the AI in two separate studies, first confirming the performance of the AI in a US cohort (sensitivity 82.2%, specificity 98.1%, negative predictive value 99.2%, c-statistic – 0.986),^29^ and second in two tertiary cardiology departments.^12^ The product (EchoSolv AS) is regulatory cleared in USA, and is intended for patients who 18 years or older who have an echocardiogram performed as part of routine clinical care.

### Method details

#### Randomised crossover study design to test the AI in clinical practice

Resting comprehensive transthoracic echocardiography (TTE) studies were randomly selected from the Picture Archive Cardiology System (PACS) of a US tertiary cardiology department (St Bernard’s Medical Center, Arkansas, United States), without knowledge of patient race or socioeconomic status. The **AI was not applied** during the process of selection of TTE studies.

##### Inclusion criteria

Male or female sex, age >21 years, reporting of presence/absence of AS, mitral regurgitation (MR) and/or left ventricular (LV) systolic dysfunction, as shown in Figure 1. Echocardiography was performed as per standard clinical workflow, with measurements performed at the time of echocardiography and the structured report (containing measurement data) transferred along with the images to the cardiology Picture Achive and Communication System (PACS). **High-gradient severe AS** was defined as a peak aortic velocity between 4.0 and 4.5 m/s and/or a mean transaortic gradient of between 40 and 45 mmHg. **Low-gradient severe AS** was defined as an AVA ≤1.0 cm^2^ and both a peak aortic velocity between <4.0 m/s and a mean transaortic gradient of between <40 mmHg. Three subtypes of low-gradient severe AS were included: **Classical low flow low gradient severe AS** had the additional characteristic of LVEF <50% and stroke volume index [SVi] < 35 mL/m^2^, whereas **paradoxical low flow low gradient severe AS** had LVEF ≥50% and stroke volume index [SVi] < 35 mL/m^2^, and **normal flow low gradient severe AS** had stroke volume index [SVi] < 35 mL/m^2^.

200 consecutive transthoracic echocardiography studies meeting the initial inclusion criteria were selected by the cardiology PACS administrator, and were fully de-identified including any burned-in patient identifiers associated with the DICOM images and SR data. These 200 echocardiograms then reviewed by a board-certified cardiologist with >20 years of experience in echocardiography. Measurements were then checked for accuracy against individual images, and consistency with the report conclusions. The selection process considered the following exclusion criteria, and if exclusion criteria were not met, the echocardiogram was included in the study. If a study was excluded, a new (de-identified) study was provided by the PACS administrator, thus 200 studies meeting inclusion and exclusion criteria were selected.

##### Exclusion criteria

Studies were excluded if image quality was insufficient to allow accurate assessment of LV systolic function, including poor endocardial definition with or without left ventricular opacification contrast (LVO). If the basic measures of aortic stenosis severity could not be determined (for example, LVOT dimension could not be accurately measured, if the Doppler trace of LVOT or aortic valve was of poor quality, or if mis-alignment of the Doppler trace was identified), the study was excluded. Similarly, if mitral regurgitation severity could not be estimated, the study was excluded. Additional exclusions included the absence of the minimum inputs required for the AI to produce an output (age, sex, height, weight, AV peak velocity and LVEF), previous aortic or mitral valve intervention, subaortic membrane or hypertrophic cardiomyopathy, or current or past infective endocarditis.

#### Independent confirmation of disease and severity

After inclusion and exclusion criteria were applied, the 200 fully de-identified echocardiograms (100 **cases** with severe AS, and 100 **controls** without severe AS but having an additional diagnosis of either ≥ moderate MR and/or ≥ moderate LV dysfunction) were provided via a cloud-based echocardiogram viewing and reporting system (Ticker Cardiology: https://www.tickercardiology.com/) to two experienced board-certified echocardiologists with over 20 years’ experience in echocardiography. These cardiologists, having no involvement with either the investigational site or Study Sponsor, did not have a clinical affiliation with each other or the initial cardiologist reviewer, and with no declared conflicts of interest, then independently interpreted each study (**before the AI was applied**), with additional adjudication by a third independent board-certified cardiologist if any disagreement in AS severity was identified. As part of this review process, an adjudication meeting was held by the three cardiologists. During these adjudication meetings, there were 8 studies where a definitive conclusion on the severity of AS was not possible due to poor image quality that had not met initial exclusions, and thus were deemed unsuitable for inclusion in the study cohort. Each of these excluded studies were replaced by another study showing the same disease process (with de-identification prior to export and sharing of studies). This study was then reviewed by the same independent cardiologists, with the process repeated until unanimous agreement on diagnosis was achieved with all three expert readers.

After final adjudication, 100 echos had severe AS (disease), 47 of which had high-gradient severe AS (peak aortic velocity between 4.0 and 4.5 m/s and/or a mean transaortic gradient of between 40 and 45 mmHg), and 53 had low gradient severe AS (AVA <1.0 cm^2^ with peak transaortic velocity <4.0 m/s and mean transaortic gradient <40 mmHg). The low-gradient severe AS groups were further sub-classified into classical low-flow low-gradient severe AS, which had the additional characteristic of LVEF <50% and stroke volume index [SVi] < 35 mL/m^2^, whereas paradoxical low flow low gradient severe AS had LVEF ≥50% and stroke volume index [SVi] < 35 mL/m^2^, and normal flow low gradient severe AS had stroke volume index [SVi] < 35 mL/m^2^. A further 100 echos without severe AS (control) were selected, 50 of whom had moderate AS and 50 with either mild or no AS, but the presence of left ventricular (LV) systolic dysfunction (LVEF <40%) and/or progressive or severe mitral regurgitation.

#### Application of AI software

The AI was then applied **after** final TTE interpretation by the expert readers. Examples of echocardiograms used in the study are shown in Figure S2, including high-gradient severe AS (Panel A and Video S1), classical low-flow low-gradient severe AS (Panel B and Video S2), paradoxical low-flow low-gradient severe AS (Panel C and Video S3), and no AS (Panel D and Video S4, an example of LV dysfunction). The corresponding parasternal long axis video loops are available in the supplementary material (see Figure S2). The AI output corresponding to each echocardiogram is shown within each Panel. The LVOT measurement error in Panel D is noteworthy in that it did not influence the AI, since LVOT data is ignored by the AI system.

Video S1. A high gradient AS PLAX This video shows high gradient severe AS with normal left ventricular systolic function viewed from the parasternal long-axis echocardiographic view.

Video S2. B cassical low flow low gradient AS PLAX This video shows classical low-flow low-gradient severe AS with impaired left ventricular systolic function viewed from the parasternal long-axis echocardiographic view.

Video S3. C paradoxical low gradient AS PLAX This video shows paradoxical low-gradient severe AS in the setting of normal left ventricular systolic function viewed from the parasternal long-axis echocardiographic view.

Video S4. D no AS PLAX This video shows no evidence of AS and impaired left ventricular systolic function viewed from the parasternal long-axis echocardiographic view.

#### Randomization

Randomization was undertaken and standard questions applied, using a web-based case-report form (CRF) were administered through an independent clinical research organization, responsible for implementing and maintaining quality assurance and written Standard Operating Procedures, with quality control provided through SMART TRIAL Clinical Data Management System (CDMS). Quality control measures were applied at each stage of data handling to ensure that data was reliable and had been processed correctly. The flow-chart of the study is shown in Figure 1.

The randomization script was developed and executed by the Electronic Data Collection (EDC) platform provided Greenlight Guru Clinical, formerly SMART TRIAL (https://www.greenlight.guru/smart-trial-is-greenlight-guru-clinical). The randomization script performed the following tasks (Figure S1).

• Extract a 50/50 split of control and disease studies and allocated to both aided (n = 100) and unaided (n = 100) groups per reader.
• Echocardiogram studies were randomized and assigned randomized ID for Session 1.
• Echocardiogram studies were then randomized again into the complimentary group originally allocated within Session 1. For example, if a study was allocated to the “Aided” group in Session 1, that echo study was then allocated to the “Unaided” group in Session 2.
• When echocardiogram studies were reallocated in Session 2, they were reassigned a new randomization ID.

#### Randomized crossover study methods

Five US board-certified cardiologists from the same tertiary hospital cardiology department in St Bernard’s Medical Center, Arkansas, United States, with no prior knowledge or exposure to the AI (to avoid any initial reporting bias), and with experience levels ranging from junior faculty (<5 years since board certification) to senior attending cardiologist, participated in the study. A sixth reader was initially involved in the study but was subsequently withdrawn by the site due to a major protocol deviation. Initial training included access to the CRF but no information or training on the AI. Each cardiologist was provided a unique hyperlink to the de-identified transthoracic echocardiogram study images, accessed via the CRF where they entered their findings after interpretation. The entire resting transthoracic study (as it was originally undertaken) was accessed by the reading cardiologist, including the measurements taken at the time of the original echocardiogram. After the first 100 unassisted reads (50 severe AS and 50 controls) according to standard workflow (review of images and measurements), each cardiologist was then provided with the study protocol, and training material, which contained information on the purpose of the study, and training including the operational characteristics and intended use of the AI. For the remaining 100 cases the results of the AI were revealed each time the CRF was accessed, and the cardiologist could choose to accept or reject the AI output during each echocardiogram interpretation. After a four-week washout period, crossover and re-randomization of the cases, the 200 studies were read again independently by each cardiologist, again using standard workflow (images and measurements) but with half also having AI assistance, ensuring that each case was read by each cardiologist, both unassisted and assisted, across the study.

The primary outcome was the improvement in the area under the receiver operating characteristic (AUROC) curve of AS diagnosis, with and without AI assistance. Secondary outcomes were the independent accuracy of the AI, human error rates, and any identified safety concerns.

This clinical study was executed in accordance with the Declaration of Helsinki, in agreement with the guidelines for conducting a clinical study in accordance with Good Clinical Practice (GCP). The clinical study and documentation including amendments were reviewed and approved by an Institutional Review Board (IRB) prior to initiation of the study. Informed patient consent was exempted due to the fully de-identified nature of the study, and that the study was considered to be of non-significant patient risk.

### Quantification and statistical analysis

The change in reader AUROC, when interpreting studies with/without AI assistance was analyzed using the Dorfman-Berbaum-Metz (DBM) method using the MRMCaov R package 0.1.3.^49^^,^^50^^,^^51^^,^^52^^,^^53^^,^^54^ This method which analyses jack-knifed pseudo-values is regularly used for MRMC studies and enables the variability in the AUROC among cases and readers to be quantified and accounted for in the focused comparison between assisted and non-assisted reads. The Receiver Operating Characteristic (ROC) curves and AUROC was then computed using the trapezoidal approximation. The difference in the AUROCs and 95% confidence interval for this difference was estimated from the DBM analysis and the corresponding two-tailed *p*-value generated. ROC curves were generated and displayed for both groups. Secondary outcomes including sensitivity, reader concordance/variability and referral recommendations between the AI-assisted and non-assisted groups were also analyzed using the DBM method.
