# Supplementary material for: A randomized crossover study of cardiologist reporting of severe aortic stenosis with/without assistance from artificial intelligence (AI)
Source: iScience. 2026 Apr 2;29(10):115562. doi: 10.1016/j.isci.2026.115562 (PMC13594739; doi:10.1016/j.isci.2026.115562)

iScience, Volume *29*

Supplemental information

A randomized crossover study of cardiologist reporting of severe aortic stenosis with/without assistance from artificial intelligence (AI)

David Playford, Dane Brescacin, Chris Frampton, Naveed Younis, and Geoff Strange

Supplementary Material

Supplementary Figure 1: Randomization Script Methodology

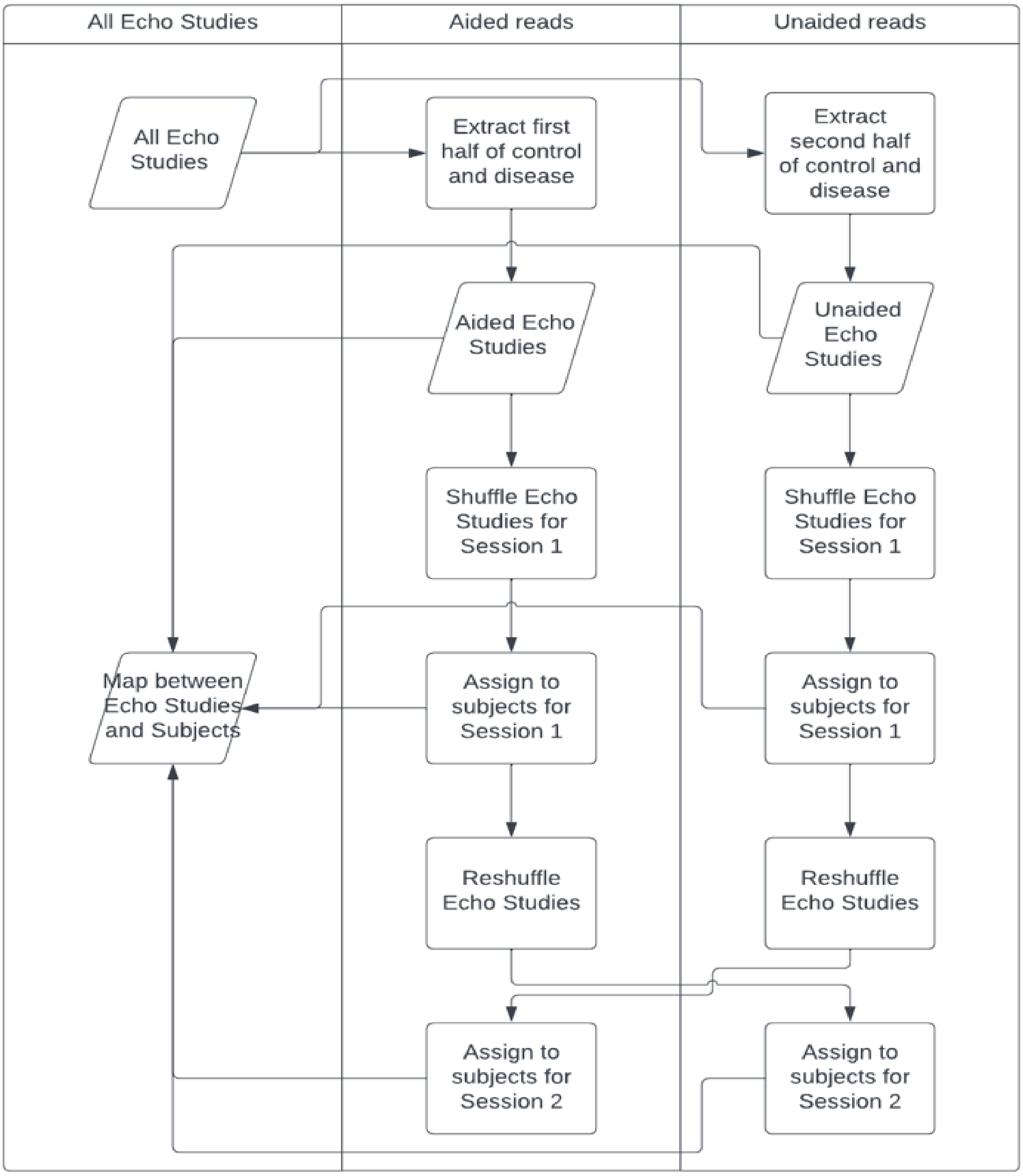

Supplementary Figure 2   Examples of echocardiograms included in the study.

Legend: Each panel shows the left ventricular outflow tract (LVOT) diameter (top left), peak and mean aortic valve velocities and gradients using continuous wave (CW) Doppler (top right), peak and mean LVOT velocities and gradients using pulsed wave (PW) Doppler (bottom right), and a screen shot of the AI output screen presented to the cardiologists when being assisted by the AI (bottom left). Panel A shows high-gradient AS severe AS; Panel B classical low-flow low-gradient severe AS (Panel B), paradoxical low-flow low-gradient severe AS (Panel C), and no AS (Panel D, an example of LV dysfunction). The LVOT measurement error in Panel D is noteworthy in that it did not influence the AI, since LVOT data is ignored by the AI system. Video loops of the parasternal long axis images corresponding to each panel are found <u>here</u> **<u>{insert hyperlink to each video}</u>**.

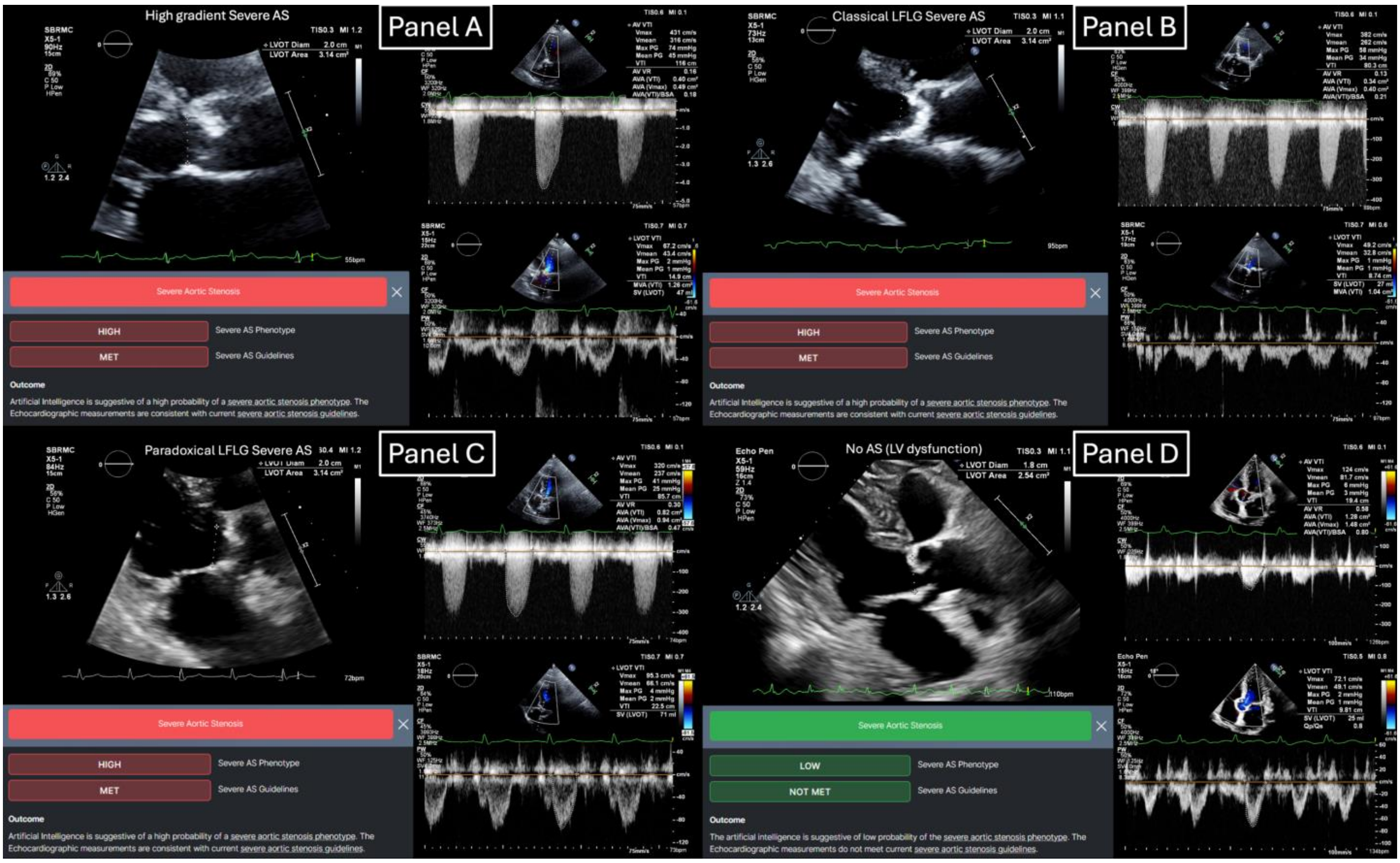

Supplement: Supplementary file 1 [file mmc1.pdf]
